# The Epstein-Barr Virus-Encoded EBNA1 Protein Activates the Bone Morphogenic Protein (BMP) Signalling Pathway to Promote Carcinoma Cell Migration

**DOI:** 10.3390/pathogens9070594

**Published:** 2020-07-21

**Authors:** Hannah E. Bridgewater, Kathryn L. Date, John D. O’Neil, Chunfang Hu, John R. Arrand, Christopher W. Dawson, Lawrence S. Young

**Affiliations:** 1 Warwick Medical School, Gibbet Hill Campus, University of Warwick, Coventry CV4 7AL, UK; H.Bridgewater.1@warwick.ac.uk (H.E.B.); C.Dawson.3@warwick.ac.uk (C.W.D.); 2 Institute for Cancer & Genomic Sciences, College of Medicine & Dentistry, University of Birmingham, Birmingham B15 2TT, UK; kathryn.date@hyms.ac.uk (K.L.D.); J.D.ONeil@bham.ac.uk (J.D.O.); C.Hu@bham.ac.uk (C.H.); j.r.arrand@bham.ac.uk (J.R.A.)

**Keywords:** Epstein-Barr virus, Epstein-Barr nuclear antigen 1, bone morphogenic protein, undifferentiated nasopharyngeal carcinoma

## Abstract

The Epstein-Barr virus (EBV)-encoded nuclear antigen 1 (EBNA1) protein is expressed in all virus-associated malignancies, where it performs an essential role in the maintenance, replication and transcription of the EBV genome. In recent years, it has become apparent that EBNA1 can also influence cellular gene transcription. Here, we demonstrate that EBNA1 is able to stimulate the expression of the Transforming growth factor-beta (TGFβ) superfamily member, bone morphogenic protein 2 (BMP2), with consequential activation of the BMP signalling pathway in carcinoma cell lines. We show that BMP pathway activation is associated with an increase in the migratory capacity of carcinoma cells, an effect that can be ablated by the BMP antagonist, Noggin. Gene expression profiling of authentic EBV-positive nasopharyngeal carcinoma (NPC) tumours revealed the consistent presence of BMP ligands, established BMP pathway effectors and putative target genes, constituting a prominent BMP “signature” in this virus-associated cancer. Our findings show that EBNA1 is the major viral-encoded protein responsible for activating the BMP signalling pathway in carcinoma cells and supports a role for this pathway in promoting cell migration and possibly, metastatic spread.

## 1. Introduction

Epstein-Barr virus (EBV) is a ubiquitous human herpesvirus that is aetiologically linked to malignancies of both lymphoid and epithelial origin, reflecting the natural tropism of the virus in vivo [1,2]. The virus exploits the B-cell differentiation programme to persist within the resting memory B-cell compartment of the immunocompetent host as a lifelong asymptomatic infection [3,4]. As a general rule, EBV establishes long-term latency in the B-lymphocyte compartment, while it is capable of replicating in both B-lymphocytes and epithelial cells [1]. However, it additionally possesses the unique ability to transform resting B-lymphocytes in vitro into continuously proliferating lymphoblastoid cell lines (LCLs) [5], indicative of its oncogenic potential. In the context of immunosuppression or other cofactors such as malaria, EBV is associated with the development of lymphomas predominantly of B-cell lineage. The aberrant establishment of latent non-replicative EBV infection in epithelial cells contributes to the development of nasopharyngeal carcinoma (NPC) and a subset of gastric carcinomas.

While the pattern of EBV latent protein expression varies in different tumour types, Epstein-Barr virus nuclear antigen 1 (EBNA1), is expressed in all EBV-associated malignancies consistent with its indispensable role in the maintenance and replication of the episomal EBV genome via sequence-specific binding to the plasmid origin of viral replication, *Ori*P [1,6]. As a DNA-binding protein, and through its ability to act as a transcriptional transactivator for certain viral promoters, EBNA1 is key to the regulation of EBNAs (including EBNA1) and latent membrane protein-1 (LMP1) [7,8,9].

The contribution of EBNA1 to the development of EBV-associated malignancies is both complex and controversial [10]. Indeed, it has been previously suggested that the expression of EBNA1 is not obligatory in B-cell transformation, as a recombinant EBV (EBV) deleted for EBNA1 retained its growth-transforming properties in the establishment of LCLs [11]. Moreover, expression of EBNA1 in Akata Burkitt’s lymphoma (BL) cells previously cleared of EBV infection was, on its own, not sufficient to confer a malignant phenotype [12]. However, direct oncogenic activity of EBNA1 in vivo has been suggested by the ability of B-cell-targeted EBNA1 expression to increase the frequency of B-cell lymphomas in transgenic mice, although this effect has been contested by later studies [13,14,15].

EBNA1 is also thought to contribute to the survival of EBV-positive Burkitt’s lymphoma (BL) cells in vitro [16], potentially through upregulation of survivin, an inhibitor of apoptosis [17]. The observed interaction of EBNA1 with the ubiquitin-specific protease, USP7, is predicted to contribute to host cell immortalisation through sequestration of USP7 and destabilisation of p53 [18,19]. This association, in addition to a distinct interaction with host CK2 kinase, elicits degradation of principal components of cellular PML nuclear bodies and potentially promotes the survival of cells harbouring DNA damage [20,21].

While EBNA1 is principally regarded as a genome maintenance protein, there is growing evidence to substantiate a role for EBNA1 in the modulation of cellular gene transcription and subsequent deregulation of key cellular processes. This has been demonstrated in the context of B-lymphocytes where EBNA1 has been shown to induce CD25 and CCL20 expression and downregulate the tumour suppressor protein, tyrosine phosphatase receptor kappa (PTPRK), in Hodgkin’s lymphoma cells [22,23,24], and to upregulate RAG1 and RAG2 expression in EBV-positive BL cells [25]. In epithelial cells, a transcriptional profiling analysis of EBNA1-expressing carcinoma cells identified the differential regulation of cellular genes involved in translation, transcription and cell signalling [26,27,28]. In addition to priming cellular responses to IFNγ through induction of STAT1, EBNA1 has been shown to modulate signalling in the TGFβ signalling pathway, increase AP-1 transcription factor activity through upregulation of c-Jun and ATF2 subunits, and abrogate NF-κB signalling through inhibition of IKKα/β phosphorylation. Additionally, it has been shown to enhance the expression of EBERs through increased cellular RNA polymerase III transcription and the induction of associated cellular transcription factors [29].

EBNA1 has already been specifically implicated in the dysregulation of TGFβ signalling in carcinoma cell lines [26]; however, its effects on the closely-related bone morphogenic protein (BMP) signalling pathway have not yet been investigated. BMP signalling represents a branch of the TGFβ superfamily most extensively characterised for its roles in embryonic development, osteoblast differentiation and subsequent bone formation [30,31,32,33], but later found to possess additional effects in diverse biological processes including cell differentiation, regulation of stem cell self-renewal and fate determination, cell growth, migration, neurogenesis, morphogenesis, apoptosis and early embryonic development [33,34,35,36,37]. Following receptor engagement at the cell surface, canonical signalling is propagated via the phosphorylation of a set of BMP-specific Smad proteins (Smad1, Smad5 and Smad9). These form active complexes with the co-Smad, Smad4, which assemble in the nucleus at promoter regions, where they recruit the necessary cofactors required for target gene transcription.

Studies are now emerging to suggest a specific function for BMPs in tumour development and progression [38,39] and, although the precise role that BMPs play in this setting remains uncertain, activation of the BMP signalling pathway, typically interpreted as increased levels of phosphorylated Smad1/5/8, has been reported in primary colon carcinoma, non-small-cell lung cancer, renal cell carcinoma and oestrogen-receptor positive and metastatic breast cancers [40,41,42,43,44]. Furthermore, overexpression of the BMP2 ligand has been documented in a variety of carcinoma-derived cell lines [45] and identified as being dysregulated in primary pancreatic cancer, oral squamous cell carcinoma and non-small-cell lung cancers [46,47,48,49]. Significantly, recent gene expression profiling of microdissected NPC tumours has identified the upregulation of the BMP2 ligand in EBV-associated NPC tumours [50]. In light of this observation, we examined the status of the BMP signalling pathway in NPC and gastric-derived carcinoma cell lines. These studies revealed, for the first time, the ability of EBNA1 to activate BMP signalling in epithelial cells, potentially via transcriptional upregulation of the BMP2 ligand.

## 2. Results

### 2.1. The BMP Signalling Pathway is Aberrantly Activated in NPC

**Figure 1:**
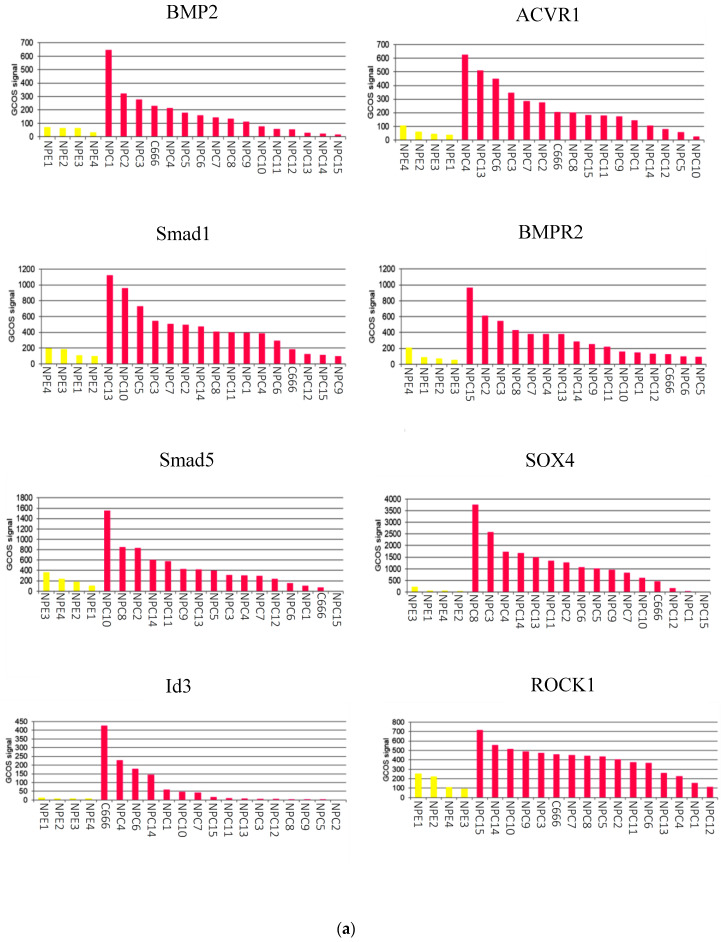

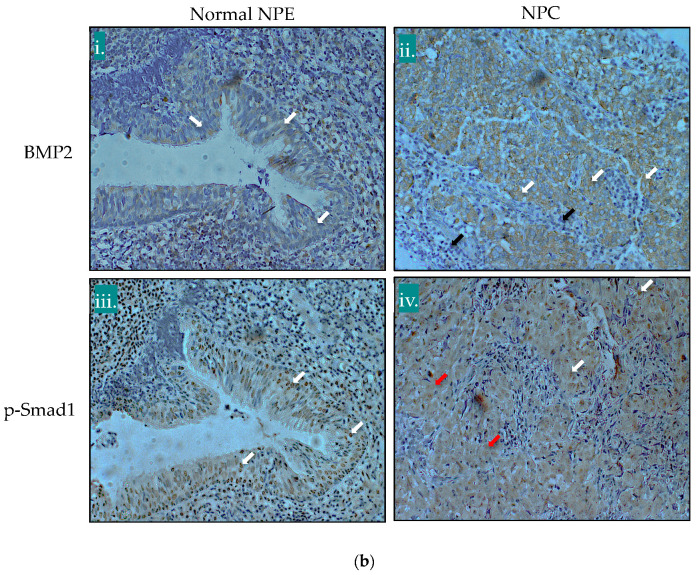
Key bone morphogenic protein (BMP) components are overexpressed in Epstein-Barr virus (EBV)-positive nasopharyngeal carcinoma (NPC). (**a**) The GeneChip^®^ Operating Software (GCOS) was used to identify significantly, differentially regulated key genes in the BMP signalling pathway using expression array data generated in a separate study from 16 tumours (pink) and 4 normal controls (yellow). (**b**) Representative immunohistochemical (IHC) staining of normal nasopharyngeal epithelium (NPE) and a single case of NPC for BMP2 (upper panels) and phosphorylated Smad1 (pSmad1) (lower panels). Upper panels: White arrows denote weak cytosolic staining of BMP2 in (i) normal nasal epithelium (NPE), compared to much stronger staining in (ii) NPC tumour cells; no staining was observed within the tumour stroma (black arrows). Lower panels: (iii) White arrows denote nuclear pSmad1 staining in normal nasal epithelium. (iv) White arrows denote strong nuclear pSmad1 and red arrows denote strong cytosolic pSmad1 staining in NPC tumour cells. Magnification x200.

To gain an overall impression of BMP pathway activity in NPC, normalised array intensities were subjected to dChip software analysis and R Studio was used to generate a heatmap displaying differentially regulated genes associated with the BMP pathway. Array intensities from 16 authentic microdissected NPC tumours along with the C666-1 NPC cell line were compared with 4 normal nasopharyngeal epithelium samples. A large number of BMP pathway components and targets were found to be upregulated relative to normal tissue, suggesting an overall activation of the BMP signalling pathway in NPC tumours and the C666-1 cell line (Figure S1, Table S1). A GeneChip^®^ Operating Software (GCOS) analysis confirmed that the overall expression of selected key BMP components (BMP2, ACVR1, Smad1, BMPR2, Smad5, SOX4, Id3 and ROCK1) was higher in EBV-positive NPC tumours than normal nasopharyngeal epithelium (Figure 1a) with average fold increases of 2.4 for BMP2 (*p*-value < 0.01), 5.2 for ACVR1 (*p*-value < 0.01), 5.1 for Smad1 (*p*-value < 0.01), 5.8 for BMPR2 (*p*-value < 0.01), 2.4 for Smad5 (*p*-value > 0.05), 14.3 for SOX4 (*p*-value > 0.05), 1.85 for Id3 (*p*-value < 0.01) and 2.24 for ROCK1 (*p*-value < 0.01) (Figure 1).

To validate these findings, immunohistochemical (IHC) staining was performed on a small series of NPC specimens (10 cases), containing adjacent normal epithelium, with antibodies specific for BMP2 and the phosphorylated “active” form of Smad1 (pSmad1). Representative IHC stains, shown in Figure 1b, revealed low levels of BMP2 staining in the cytosol of normal nasopharyngeal epithelium (panel (i), white arrows), while in NPC, significantly higher levels of BMP2 were observed in the cytosol of tumour cells (panel (ii), white arrows); stromal tissue failed to stain for BMP2 (panel (ii), black arrows). Although nuclear pSmad1 was observed in the normal epithelium (panel (iii), white arrows), the expression of both nuclear (panel (iv), white arrows) and cytosolic pSmad1 (panel (iv), red arrows) was more extensive in NPC tumour cells. Quantitation of IHC staining between NPC tumours and normal nasal epithelium revealed average intensity scores of 6.1 versus 3.6 (*p*-value < 0.0001) for BMP2, and 7.7 versus 4.4 (*p*-value < 0.001) for pSmad1.

### 2.2. The EBV-Positive NPC Cell Line, C666-1, Shows Constitutive BMP Pathway Activity

**Figure 2:**
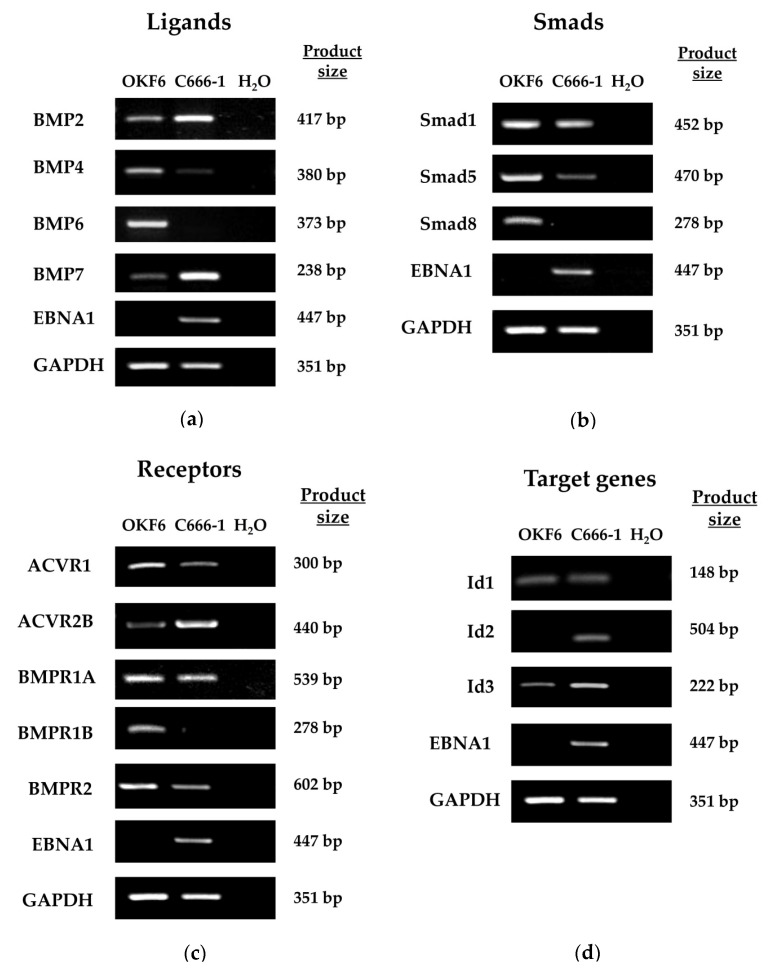
Expression of BMP pathway components in OKF6 and C666-1 cells. RT-PCR analysis for mRNA expression levels of (**a**) BMP ligands (BMP2, 4, 6 and 7), (**b**) BMP-specific Smads (Smad1, Smad5 and Smad8), (**c**) BMP receptors (ACVR1, ACVR2B, BMPR1A, BMPR1B and BMPR2 and (**d**) classical BMP target genes (Id1, Id2 and Id3) in OKF6 and C666-1 cells. Epstein-Barr virus nuclear antigen 1 (EBNA1) was included as a control for EBV expression in C666-1 cells and GAPDH was included as a positive control to confirm equal RNA input into the PCR reactions, while negative water controls confirmed the absence of contamination.

To expand upon findings from the gene expression profiling analysis, comparisons were made between the authentic EBV-positive NPC cell line, C666-1, and OKF6, a telomerase-immortalised oral keratinocyte cell line; the latter was used as a suitable control given its sensitivity to TGFβ1-mediated cell growth suppression [51]. RT-PCR analysis was used to profile key components of the BMP pathway to determine which genes are specifically altered by the presence of EBV (Figure 2).

Several BMP ligands were examined and, in addition to confirming the increase of BMP2 expression in the C666-1 NPC cell line, this analysis also identified a substantial upregulation of BMP7 at the mRNA level. Conversely, both BMP4 and BMP6 appeared to be transcriptionally downregulated in C666-1 cells. 

BMP ligands are reported to bind a variety of type I and type II receptors. The upregulation of ACVR2B was found in the C666-1 cell line as compared to the OKF6 control, with little change in the expression of BMPR1A, but downregulation of ACVR1, BMPR1B and BMPR2 (Figure 2). These receptors recruit and specifically activate the BMP-specific R-Smads, Smad1, Smad5 and Smad8, to propagate the BMP signal. The expression of Smad1 was relatively unchanged between OKF6 and C666-1 cells, while Smad5 expression was decreased and Smad8 could not be detected in C666-1 cells (Figure 2).

**Figure 3:**
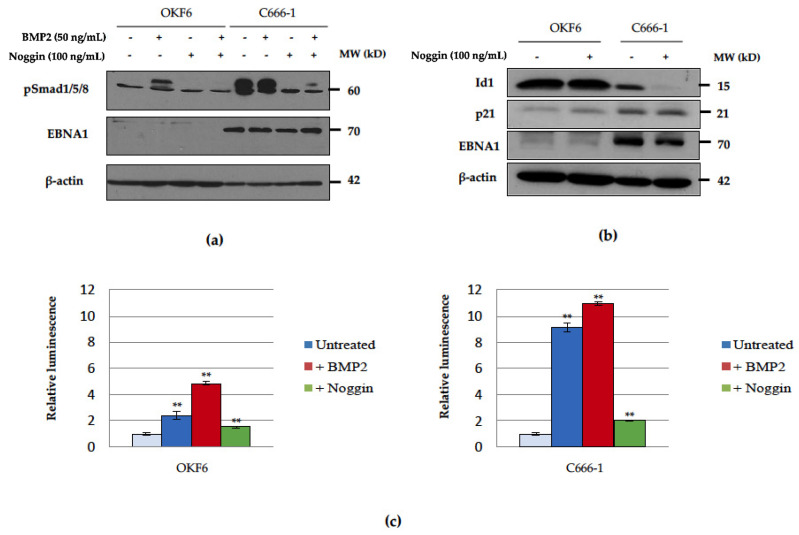
Basal levels of Smad1/5/8 phosphorylation are elevated in the EBV-positive C666-1 NPC cell line. (**a**) Immunoblotting for protein expression levels of the phosphorylated form of Smad1/5/8 in serum-starved OKF6 and C666-1 cells treated with 100 ng/mL recombinant Noggin for 1 h, followed by 50 ng/mL recombinant hBMP2 for a further 24 h, or left unstimulated as controls. β-Actin was included to confirm equal protein loading. (**b**) Representative immunoblot showing a reduction in Id1 expression in C666-1 cells following treatment with 100 ng/mL Noggin for 24 h. β-Actin was included to confirm equal protein loading. (**c**) BRE-Luc reporter assays showing basal and stimulated BRE-Luc activity in OKF6 and C666-1 cells following treatment with 50 ng/mL BMP2 or 100 ng/mL Noggin for 24 h (±SD) (** denotes a *p*-value < 0.01 and * denotes a *p*-value < 0.05).

In several studies, BMP treatment has identified the Id genes, Id1, Id2 and Id3, as direct BMP target genes [52,53,54,55]. An examination of their relative expression levels in OKF6 and C666-1 cells revealed little difference between the two cell lines in terms of Id1 expression, but increased expression of both Id2 and Id3 in C666-1 cells (Figure 2). BMP pathway activation was assessed in OKF6 and C666-1 cells by examining the relative levels of phosphorylated Smad1/5/8. Immunoblotting of total cell lysates after treatment with BMP2 showed that the basal levels of phosphorylated Smad1/5/8 were substantially higher in C666-1 cells compared with OKF6 cells and were blocked by the BMP inhibitor, Noggin (Figure 3a). Stimulation with BMP2 resulted in induction of Smad1/5/8 phosphorylation in OKF6 cells but did not further potentiate the constitutively raised levels observed in C666-1 cells, perhaps indicating that the BMP signalling pathway was already saturated in this cell line (Figure 3a). The BMP antagonist, Noggin, was effective in reducing the levels of Smad1/5/8 phosphorylation, which were partially restored by the addition of BMP2 in C666-1 cells, but not in OKF6 cells (Figure 3a). 

Having established that the BMP pathway is constitutively activated in C666-1 cells, we sought to determine whether disruption of the pathway would impact BMP-mediated gene expression. BMP2 has been implicated in the induction of p21 and Id1 expression [56]. As shown in Figure 3b, the addition of the BMP antagonist, Noggin, had little detectable effect on the expression level of Id1 protein in OKF6 cells, which was basally much higher than that detected in C666-1 cells. In contrast, Id1 expression in C666-1 cells was completely abolished in response to BMP inhibition with Noggin (Figure 3b). The expression of p21, however, was relatively unaffected by interfering with BMP signalling in both cell lines studied. 

#### BRE-Luciferase Reporter Activity is Elevated in the EBV-Positive C666-1 Cell Line

The integrity of the BMP signalling pathway was further investigated using a BMP-specific reporter construct, BRE-Luc, which contains a BMP-response element (BRE) from the mouse Id1 promoter. The BRE-luciferase reporter construct is specifically activated by BMP, but not by either TGFβ or activin [52]. OKF6 and C666-1 cells were transiently transfected with the BRE-Luc reporter and were subsequently either stimulated with 50 ng/mL BMP2 or incubated with 100 ng/mL of the BMP antagonist, Noggin, for 24 h. Relative luciferase activity was determined, and is depicted graphically in Figure 3c. Stimulation with BMP2 was sufficient to induce a 2-fold increase in the levels of BRE-Luc reporter activity in OKF6 cells, while the addition of BMP2 had only a modest effect on the measured levels of BRE-Luc reporter activity in C666-1 cells, likely reflecting saturation of the BRE by the very high endogenous levels of activated pSmad1/5/8 in the NPC cell line. Noggin was effective in significantly inhibiting BRE-Luc activity in both cell lines with a profound effect on the constitutively high levels of BMP activation in C666-1 cells (Figure 3c).

### 2.3. Gene Expression Profiling of BMP Pathway-Associated Genes in EBNA1-Expressing Ad/AH Cells

**Figure 4:**
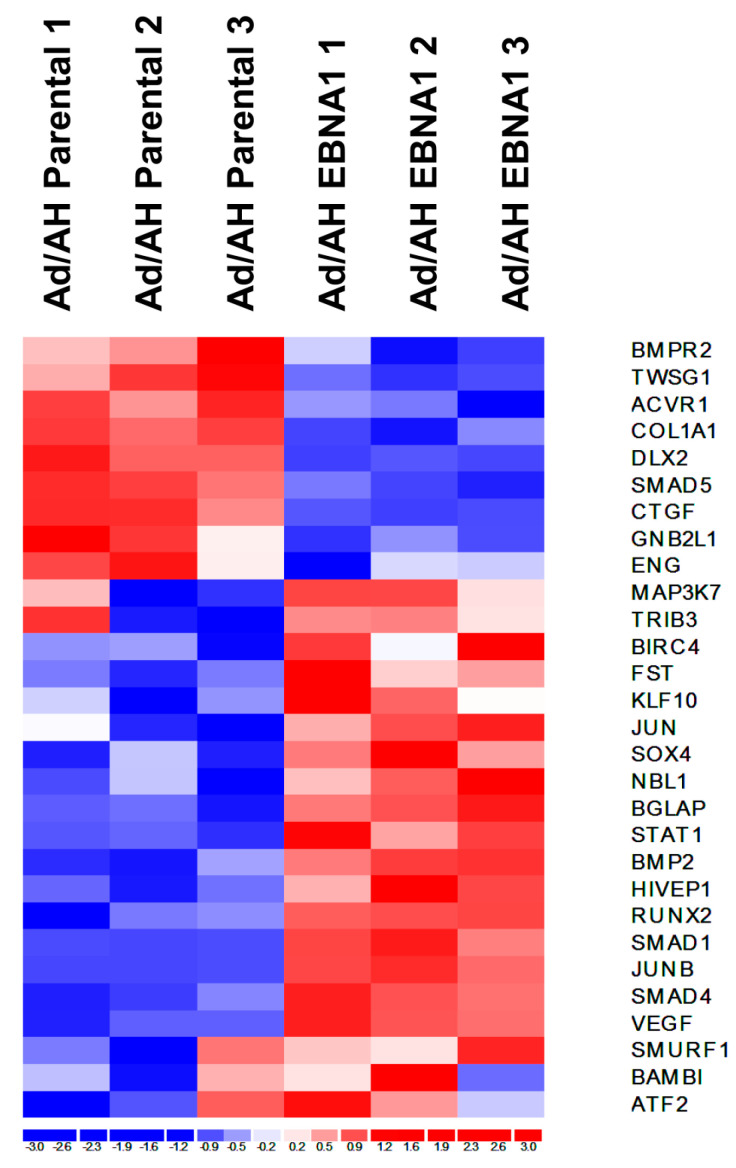
Gene expression profiling of BMP-associated genes in Ad/AH cell lines stably expressing EBNA1. Extensive literature searches revealed a set of genes associated with the BMP signalling pathway. The resultant gene list was utilised to identify significantly, differentially regulated genes, using expression array data generated in a separate study from Ad/AH cells stably expressing EBNA1 and parental Ad/AH cells, which were visualised using the dChip software for hierarchical clustering analysis. The expression level of each gene in an individual sample is colour-coded: blue for downregulation, red for upregulation and white for unchanged.

To ascertain whether the effects of EBV on BMP signalling were due to the virus-encoded EBNA1 protein, an overall view of differentially regulated BMP pathway-associated genes was obtained through a hierarchical clustering analysis of existing Affymetrix gene expression array data obtained from EBV-negative Ad/AH carcinoma cells stably expressing EBNA1 versus the parental Ad/AH cell line [28]. The resultant heat map of differentially regulated genes generated using the dChip software is shown in Figure 4. A large number of upregulated genes (red) implicated EBNA1 in the activation of the BMP signalling pathway.

### 2.4. Expression of BMP2 Ligand Is Increased in Ad/AH, HONE-1 and AGS Cells Expressing EBNA1 or Latently Infected with EBV

Given the reported upregulation of BMP2 in NPC tumour samples [50] and our current observations on elevated BMP2 expression in C666-1 cells, we chose to examine the basal levels of BMP2 expression in a panel of EBNA1-expressing cell lines as compared with cells latently infected with EBV. To this end, stable EBV-infected and EBNA1-expressing clones were generated in the Ad/AH, HONE-1 and AGS backgrounds, alongside neomycin-resistant controls. RT-PCR analysis confirmed the expression of EBNA1 in the stable EBNA1-transfected and EBV-infected clones of each cell line, and its absence in the neomycin-resistant control clones (Figure S2). To confirm the expression of EBNA1 at the protein level, total cell lysates were subjected to immunoblotting with K67, an antiserum specific for EBNA1. Representative immunoblots (Figure S2) confirmed the expression of EBNA1 in EBNA1-transfected cells, with levels that were broadly similar to those observed in EBV-infected cell clones of the same cell panel.

**Figure 5:**
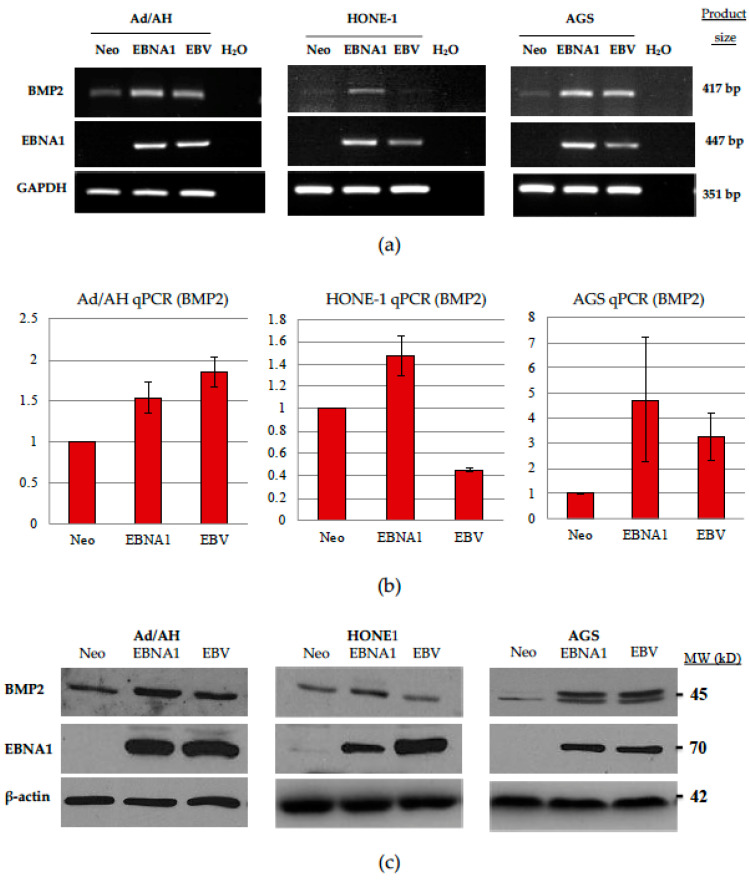
Expression of BMP2 is increased in Ad/AH, HONE-1 and AGS carcinoma cell lines expressing EBNA1 or latently infected with EBV. (**a**) RT-PCR analysis of levels of BMP2 mRNA expression in Ad/AH, HONE-1 and AGS cells expressing either a neomycin resistance cassette or EBNA1, or stably infected with EBV. GAPDH was included as a positive control to confirm equal RNA input into the PCR reactions, while water alone confirmed the absence of contamination. (**b**) qPCR analysis for BMP2 mRNA levels is also shown, in which GAPDH was included as an internal baseline control. Histograms are shown displaying the mean fold change differences ± SE (*n* = 3) relative to neomycin control cells (** denotes a *p*-value < 0.01 and * denotes a *p*-value < 0.05). (**c**) Immunoblotting for BMP2 protein levels in Ad/AH, HONE-1 and AGS cells expressing a neomycin resistance cassette or EBNA1, or stably infected with EBV, with β-actin included to confirm equal protein loading.

RT-PCR was then performed to examine baseline BMP2 expression in control, EBNA1-expressing and EBV-infected cells. As shown in Figure 5a, BMP2 transcription was significantly increased in EBNA1-expressing cells across all three epithelial cell lines. This increase was evident in EBV-infected Ad/AH and AGS cells but could not be shown for EBV-infected HONE-1 cells. These same differences were confirmed and quantified by qPCR (Figure 5b), which demonstrated an approximately 1.5-fold increase in expression, attributable to the presence of EBNA1 in Ad/AH and HONE-1 cell lines, with more pronounced increases in the AGS-EBNA1 cell line, in relation to their respective neomycin (Neo)-resistant control counterparts. Protein lysates were subsequently generated, and immunoblotting was performed to determine the relative levels of BMP2 protein. Levels of BMP2 protein were increased in EBNA1-expressing and EBV-infected cell lines, where corresponding increases in BMP2 transcripts were observed (Figure 5c). 

### 2.5. Expression of BMP Pathway Components in Ad/AH, HONE-1 and AGS Cell Lines

An RT-PCR analysis was used to determine if EBV affects the expression of key components of the BMP pathway. The mRNA expression levels of a selection of type I and type II BMP receptors, in addition to the BMP-specific Smads, Smad1, Smad5 and Smad9, were examined in Ad/AH, HONE-1 and AGS cells expressing either a neomycin resistance cassette or EBNA1, or stably infected with EBV. As shown in Figure S3, the majority of pathway components were expressed in all cell lines with the exception of the AGS cell line which, regardless of EBNA1 expression status, lacked detectable expression of BMPR1B and Smad9 transcripts. The most notable findings were the downregulation of Smad9 in both EBNA1-expressing and EBV-infected Ad/AH and HONE-1 cells, and the consistent downregulation of BMPR2 in the presence of EBNA1 and EBV across all three cell lines.

### 2.6. Basal Unstimulated Levels of Phosphorylated Smad1/5/8 Are Increased in the Presence of EBNA1 and EBV

**Figure 6:**
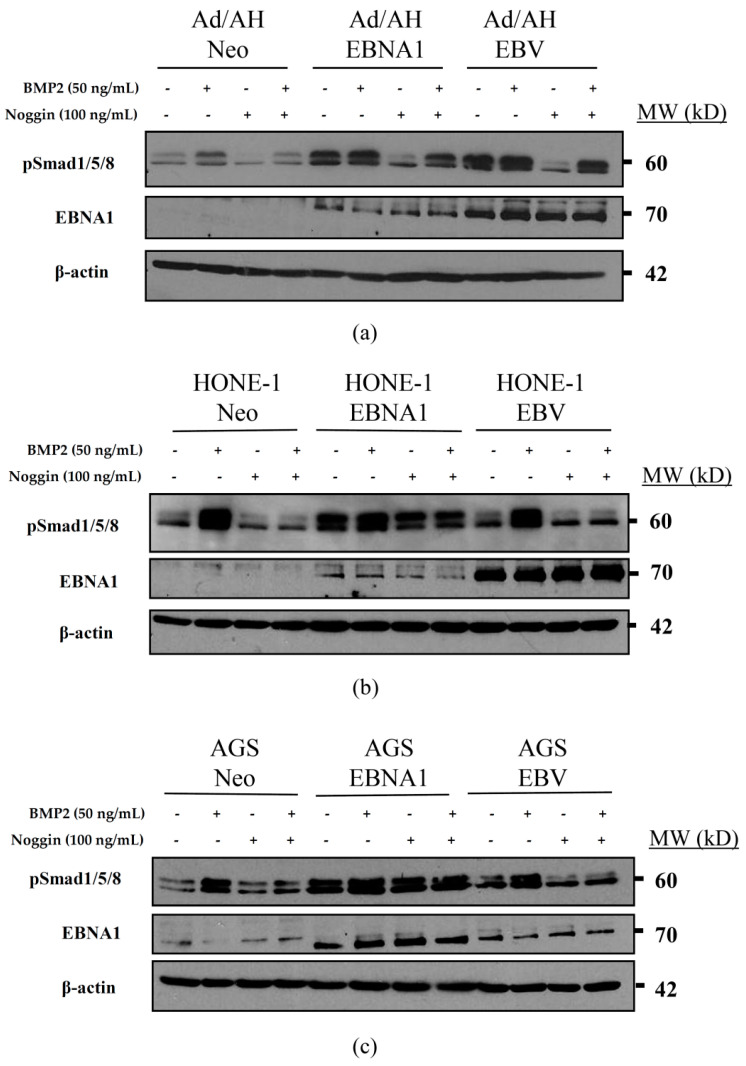
Basal expression of phosphorylated Smad1/5/8 (pSmad1/5/8) in control, EBNA1 expressing and EBV infected Ad/AH, HONE-1 and AGS cell lines. Immunoblotting for protein expression levels of the phosphorylated form of Smad1/5/8 (pSmad1/5/8) in serum-starved (**a**) Ad/AH, (**b**) HONE-1 and (**c**) AGS cells expressing either a neomycin resistance cassette or EBNA1, or stably infected with EBV, and treated with 100 ng/mL recombinant Noggin for 1 h, then subsequently with 50 ng/mL recombinant BMP2 for a further 24 h. Blots were re-probed for β-actin to confirm equal protein loading.

As activation of the BMP pathway results in phosphorylation of Smad1/5/8, the levels of phosphorylated Smad1/5/8 were determined in Ad/AH, HONE-1 and AGS Neo control, EBNA1 and EBV-infected cells by immunoblotting using an antiserum specific for the phosphorylated forms of Smad1/5/8. As shown in Figure 6, the basal levels of phosphorylated Smad1/5/8 were elevated in EBNA1-expressing cells across all three cell lines, and it was especially evident in the EBV-infected Ad/AH cell line (Figure 6a). However, unlike their Neo control counterparts, the phosphorylation of Smad1/5/8 in EBNA1-expressing cells was not further induced by BMP2 stimulation. Treatment with the natural BMP antagonist, Noggin, suppressed the levels of Smad1/5/8 phosphorylation, which, in the Ad/AH EBNA1-expressing and EBV-infected cell lines, were regained by subsequent BMP2 stimulation. However, Noggin treatment had little effect on the levels of Smad1/5/8 phosphorylation in HONE-1 and AGS EBNA1-expressing cells (Figure 6b,c).

### 2.7. EBNA1 Induction of BMP2 Promotes Cell Migration, an Effect That Can Be Reduced by the Addition of the Natural BMP Antagonist, Noggin

**Figure 7:**
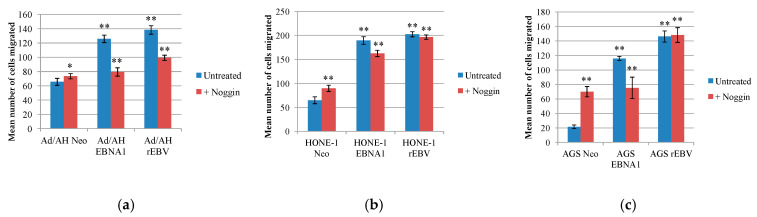
The effect of inhibition of BMP signalling on the migration of control, EBNA1-expressing and EBV-infected Ad/AH, HONE-1 and AGS cell lines. Serum-starved (**a**) Ad/AH, (**b**) HONE-1 and (**c**) AGS cells expressing either a neomycin resistance cassette (Neo control) or EBNA1, or stably infected with EBV were seeded into the upper wells of transwell migration chambers in serum-free medium with and without 100 ng/mL Noggin and allowed to migrate for 24 h. The number of migrated cells in five representative fields were counted and histograms were created showing the mean number of cells migrated (±SD) (** denotes a *p*-value < 0.01 and * denotes a *p*-value < 0.05).

BMP2 has been specifically associated with the migratory and invasive capacity of prostate and liver cancer cells in vitro [57,58,59], while the BMP antagonist, Noggin, inhibits the migration of BMP2-stimulated prostate cancer cells [60,61]. The effect of BMP pathway inhibition on the migration of Ad/AH, HONE-1 and AGS cell panels was similarly measured by a transwell migration assay. Representative results from five experiments are shown in Figure 7 and the original photomicrographs for each cell line in one experiment are shown in Figure S4. Compared with Neo control cells, both EBNA1-expressing cells and their EBV-infected counterparts displayed significantly higher rates of migration on fibronectin over a 16-h period. In the HONE-1 and AGS cell lines, treatment of Neo control cells with Noggin had a marked positive effect on migration, suggesting that BMP signalling in these cells, albeit at a low extent, is tumour-suppressive. In contrast, cell migration was partially inhibited by Noggin treatment in EBNA1-expressing cells, but not their EBV-infected counterparts (Figure 7b,c). In Ad/AH cells, however, Noggin was effective in causing partial inhibition of the migration of both EBNA1-expressing and EBV-infected cells, with a comparatively negligible effect in the Neo control cells (Figure 7a).

## 3. Discussion

In this study, we show for the first time that EBV latent infection of carcinoma cells results in the induction of bone morphogenic proteins (BMPs) with constitutive activation of the BMP pathway. More specifically, we demonstrate that expression of the EBV-encoded EBNA1 protein alone is sufficient to elicit these effects. This observation complements our previously published data describing elevated expression of components of the TGFβ/activin and BMP pathways in the EBV-positive NPC cell line, C666-1, and authentic EBV-positive NPC tumours [50]. Our findings further implicate TGFβ/BMP pathway dysregulation in the aetiology of EBV-associated cancers and highlight the contribution of a viral-encoded protein in this effect. While several reports have implicated retinoids, Sp1 transcriptional factors and the protein kinase C pathway in the transcriptional regulation of BMP2 [62,63,64], perhaps of most interest is the reported upregulation of BMP2 by FGF2, and the mediation of this effect by Runx2 [65]. A microarray analysis revealed that two factors known to regulate BMP2 expression, FGF2 and Runx2, were significantly upregulated in response to EBNA1 expression in Ad/AH cells, with 1.69- and 2.97-fold changes, respectively. However, further validation is required to establish whether the upregulation of FGF2 and Runx2 is linked to BMP2 expression in EBV-associated NPC and other virus-associated tumours. Profiling the carcinoma cell line panel for BMP receptors and key pathway components confirmed the overall integrity of the pathway in all three cell lines studied, indicating their capacity to respond to BMP ligand stimulation. Indeed, measurement of Smad1/5/8 phosphorylation and BRE-Luc reporter activity confirmed the functional integrity of the BMP signalling response in all cell lines. The increased levels of basal phosphorylated Smad1/5/8 in both EBV-infected and EBNA1-expressing cells, support a role for EBV in modulating BMP signalling through autocrine induction of BMP ligand(s).

Results from our gene expression profiling study are in broad agreement with previous studies performed on non-small-cell lung cancer (NSCLC) and head and neck squamous cell carcinoma (HNSCC), where greater than 95% of cases have been shown to overexpress BMP2, phosphorylated SMAD1/5, and Id1 [48,66]. Moreover, in metastatic HNSCC, cancer-containing lymph nodes have been shown to express high levels of BMP (BMP2, 4 and 5), highlighting a role for the pathway in metastatic spread [48,67]. One other study has shown that high levels of BMP2 are also associated with disease relapse and increased rates of local recurrence in HNSCC [66]. Given the prominent role of BMPs in bone formation, it is not surprising that bone is a common site of metastasis in prostate, lung and breast cancers [67] and that BMPs have been linked to the acceleration of bone metastasis in these tumours [44,57,58,68,69]. It is therefore feasible that the enhanced BMP signalling activity we have found in NPC may specifically contribute to bone metastasis, which is associated with more advanced cases and with poor prognosis in this tumour [70].

As with TGFβ, the roles played by BMPs in cancer are complex as demonstrated by their dichotomous effects in different cancer types and at different stages of malignant progression. Numerous studies have shown that BMP signalling is maintained in certain tumours such as breast, lung and HNSCC, where tumours overexpress BMP ligands and upregulate the BMP receptors (e.g., BMPR1A). In these situations, BMP signalling contributes to tumour growth, enhances cell survival and endows cells with motile properties through the generation of epithelial-to-mesenchymal transition (EMT). In authentic NPC tumours, expression of BMPR1A was increased relative to normal nasal epithelium. This phenomenon may be linked to malignant progression given that overexpression of BMPR1A has been implicated in the malignant transformation of oral squamous cell carcinoma [47]. As BMPR1A was proposed to convey a stimulatory effect on prostate cancer cell growth specifically [44,71], its increased expression may serve to fulfil similar roles in the progression of EBV-associated epithelial malignancies.

In contrast to the lung, breast and HNSCC, other cancer types downregulate the BMP pathway in much the same way as has been described for TGFβ. This can occur through a variety of mechanisms, which may involve a loss-of-function mutation or epigenetic silencing of BMPR2 [72,73,74], or through paracrine induction of BMP antagonists such as Gremlin on tumour-associated stromal cells [75]. One particularly striking result was the downregulation of BMPR2 in cells expressing EBNA1 or infected with EBV. This was in contrast to the NPC cell line, C666-1, and authentic NPC tumours, where BMPR2 expression was maintained. Loss-of-function mutations in BMPR2 have been implicated in the development of primary pulmonary hypertension [76], while the loss of BMPR2 expression appears to be a frequent event in cancer progression, as has been observed, particularly in metastatic variants, in renal cell carcinoma, prostate cancer and bladder cancer [72,73,74,77]. In support of this, the expression of a dominant-negative BMPR2 potentiated tumour cell proliferation and lung metastases in a mouse model of mammary carcinoma formation [78], suggesting that BMPRII loss or silencing is probably cell line- or tissue type-specific.

Poorly differentiated prostate cancers are commonly found to exhibit a loss of expression of at least one BMP receptor [79], a strategy that may provide tumour cells with a mechanism for ablating specific BMP-mediated growth-inhibitory signalling responses, while retaining other more favourable effects. Moreover, a loss of expression of the BMPR2 receptor would unlikely have such a profound impact as, for example, a loss of TGFBR2 on TGFβ signalling, as BMPs can also signal through the activin receptors, ACVR2A and ACVR2B, which may adequately compensate in the transmission of BMP signals. Indeed, deletion of BMPR2 in pulmonary artery smooth muscle cells in an ex vivo system failed to abolish BMP signalling [76], with cells alternatively transmitting signals via ACVR2A. Type II receptors do, however, differ in their ability to interact with type I receptors, and the resulting receptor complexes formed, therefore, vary in signalling capacity. The mechanism responsible for the downregulation of BMPR2 in EBNA1-expressing and EBV-infected carcinoma cells in vitro is unclear, but the fact that these cells showed robust Smad1/5/8 phosphorylation suggests that ACVR2A or ACVR2B can substitute for BMPR2. The exact nature of the effects of this shift in receptor usage is, therefore, worthy of further investigation.

The addition of exogenous BMP2 failed to elevate the levels of pSmad1/5/8 in EBNA1-expressing carcinoma cell lines further, suggesting that pathway activation had become saturated in these cells. In EBNA1-expressing and EBV-infected Ad/AH cells, inhibition of Smad1/5/8 phosphorylation in response to Noggin treatment could be effectively restored to original levels by the subsequent exogenous addition of BMP2, indicating that BMP2 is the predominant contributor to Smad1/5/8 phosphorylation in these cells. However, in the EBV-infected HONE-1 and AGS cell lines, stimulation with BMP2 was not sufficient to restore levels of Smad activation. In these cell lines, other BMP ligand subtypes may play a more influential role. Indeed, a study comparing osteoinductive and non-osteoinductive strains of osteosarcoma cells concluded that the potential for osteoinductivity was likely conferred by the specific complement of BMP subtypes present, their threshold levels and interactions with each other [80]. More comprehensive profiling of BMP ligand expression in EBV-infected epithelial cells in vivo and in vitro is required to gain further insight.

Interestingly, in EBNA1-expressing HONE-1 and AGS cells, unlike their Neo control and EBV-infected counterparts, the BMP antagonist, Noggin, proved ineffectual in diminishing the levels of phosphorylated Smad1/5/8. The effectors responsible for the phosphorylation of Smad1/5/8 in these cell lines may be BMP ligands that are not targeted by Noggin. However, Noggin still retained the ability to diminish BRE-Luc reporter activity in these same cell lines. Alternatively, a significant contribution to the measured levels of phosphorylated Smad1/5/8 may instead be the result of TGFβ-mediated phosphorylation. In support of this, the addition of exogenous TGFβ1 was found to increase Smad1/5/8 phosphorylation in OKF6 cells and smaller effects on Ad/Ah, HONE-1 and AGS cells. TGFβ stimulation did not affect C666-1 cells due to the lack of TGFBR2 expression [50] (Figure S5).

The effects of BMPs in the epithelial cell lines may parallel those observed with TGFβ, uncoupling and acquiring resistance to potentially tumour-suppressive effects, while allowing pro-tumourigenic response to predominate [39]. An example of one such response is enhanced cellular migration in the presence of EBNA1 and EBV, as assessed by the transwell migration assay. The results also suggest that increased BMP signalling within EBNA1-expressing carcinoma cell lines, as well as in EBV-infected Ad/AH cells, contributed to this increase in cell migration, as it was susceptible to reduction by Noggin. However, Noggin treatment did not markedly reduce the migration of EBV-infected HONE-1 and AGS cells. In these same cell lines, Smad1/5/8 was not constitutively phosphorylated as in the EBNA1-expressing cells, and BRE-Luc activity was also reduced in relation to control cells. This suggests that the expression of additional latent EBV gene products in these cell lines, but not in the Ad/AH cell line, has an inhibitory effect on BMP signalling. 

Increased expression of the cellular microRNA, miR-155, has been shown to have an inhibitory effect on BMP signalling, specifically inhibiting the expression of Smad1 and Smad5, by targeting their 3′ untranslated regions [81,82]. EBV infection has been shown to induce the expression miR-155 in primary B-lymphocytes [83], and upregulation of miR-155 has also been documented in NPC cell lines and tumour samples [84,85]. In B-cells, this effect has been correlated with the expression of EBNA2 and LMP1 [83,86,87], while in NPC cells it is thought to be driven, at least in part, by the concerted actions of LMP1 and LMP2A [85]. However, this is not a global effect in epithelial cells, as EBV infection failed to induce miR-155 expression in HEK293 and HeLa cells [83]. It is, therefore, possible that the expression of additional virus genes in the EBV-infected lines may serve to inhibit BMP signalling in the HONE-1 and AGS lines, but not in the Ad/AH line. It is possible that HONE-1 and AGS cells express higher levels of miR-155, but this needs to be confirmed in subsequent studies. High levels of Smad9 expression, which appears not to be targeted by miR-155 [82], could partly compensate for the effects on the BMP pathway, allowing cells to elicit appropriate responses to BMP signalling even in the presence of miR-155. This may be especially relevant in the AGS cells, where expression of Smad9 mRNA was not detected.

While at present, the interaction of viral proteins with the BMP signalling pathway has not been widely examined in the context of virus-associated malignancies, one study has reported the ability of the hepatitis B virus-encoded oncoprotein pX to activate BMP2-induced transcription in vivo, providing a potential contributory factor to hepatitis B virus-induced liver fibrosis [88]. More recently, the Kaposi’s sarcoma herpes virus (KSHV)-encoded nuclear protein, LANA, a functional homologue of EBNA1, has been shown to bind SMAD1 and to induce BMP signals in the absence of a ligand. Interestingly, the induction of Id1, a BMP-SMAD1 target protein, was shown to be important for LANA-mediated transformation of endothelial cell lines [89]. Our data add to the general phenomenon that the oncogenic γ-herpesviruses, EBV and KSHV, usurp the BMP-SMAD signalling pathway to promote cell transformation and enhance cell motility. Our study demonstrates that EBNA1, like LANA, may commandeer specific facets of BMP signalling to advance the progression of EBV-associated malignancies providing an interesting potential target for therapeutic intervention.

## 4. Materials and Methods

### 4.1. Cell Lines and Tissue Culture

Ad/AH (a human adenocarcinoma cell line derived from the nasopharynx), HONE-1 (an EBV-negative NPC cell line), AGS (a human gastric carcinoma-derived cell line) and C666-1 cell lines were cultured in RPMI medium 1640 supplemented with 8% foetal calf serum (FCS), 2 mM glutamine and 1% penicillin-streptomycin solution (Sigma-Aldrich, St Louis, MO, USA). Cells stably expressing EBNA1 and stably infected with EBV were generated as described previously [26,51].

Recombinant human BMP2 and Noggin (PeproTech, London, UK) were added to serum-starved cells in serum-free growth medium at a concentration of 50 ng/mL and 100 ng/mL, respectively.

### 4.2. RT-PCR and Real-Time Quantitative PCR (qPCR)

RNA was extracted using the EZ-RNA total RNA isolation kit (Geneflow, Lichfield, UK) and was reverse transcribed with Superscript III (Invitrogen, Carlsbad, CA, USA) following the manufacturer’s protocol, using random primers (Promega, Madison, WI, USA). RT-PCR was carried out on resultant cDNA using standard procedures with the primers listed in Table 1. Products were visualised on 1% agarose gels.

### 4.3. GCOS Analysis of Gene Expression Data

Existing expression array data obtained from 4 normal nasopharyngeal epithelium and 15 NPC tissue samples [50] were analysed using the GCOS software package (Affymetrix, Santa Clara, CA, USA). Data was probed for the expression levels of genes of interest. Normalised expression array intensities for 4 normal samples (yellow) and 15 NPC tumours plus the C666-1 EBV-positive NPC cell line (pink) are shown. 

qPCR for BMP2 was performed using a readily synthesised Taqman Gene Expression primer and probe (FAM-labelled) mix (Hs00154192_m1; Applied Biosystems, Foster City, CA, USA), and a huGAPDH primer/probe set was included as an internal baseline control. Reactions were performed in biological and technical triplicates following standard procedures, and analysed on an ABI 7500 Fast real-time PCR machine. Data were analysed using the 2^−ΔΔCt^ method [52].

### 4.4. Western Immunoblotting

Subconfluent cultures were lysed in RIPA buffer, before separation on polyacrylamide gels and transfer of proteins to nitrocellulose membranes. After blocking, membranes were probed with primary antibodies to detect EBNA1 (rabbit K67, 1:1000; a kind gift from Professor Jaap Middeldorp, Amsterdam, UMC), β-actin (mouse 1:10,000; Abcam, Cambridge, UK) BMP2 (rabbit 1:1000; Abcam, Cambridge, UK), phospho-Smad1 (Ser463/465)/-Smad5 (Ser463/465)/-Smad8 (Ser426/428) (rabbit 1:1000; Cell Signalling Technology, Danvers, MA, USA) and phospho-Smad2 Ser465/467 (rabbit 1:1000; Cell Signalling Technology, Danvers, MA, USA). The bound antibody was then detected using the appropriate anti-mouse or anti-rabbit horseradish peroxidase-conjugated secondary antibody (Dako), Glostrup, Denmark and visualised by enhanced chemiluminescence.

### 4.5. Luciferase Reporter Assays

For luciferase assays, the dual-luciferase reporter assay (Promega, Madison, WI, USA) was performed according to the manufacturer’s instructions. Cells were transiently transfected with either pGL3-basic or BRE-Luc, a BMP-specific reporter plasmid containing a BMP-response element (BRE) from the mouse Id1 promoter [52] (provided by Dr Peter ten Dijke, Leiden University Medical Centre) using Lipofectamine 2000 (Invitrogen, Carlsbad, CA, USA). To normalise transfection efficiencies, a TK promoter-driven *Renilla* luciferase plasmid (Promega, Madison, WI, USA) was co-transfected as an internal control. All assays were carried out in triplicate and represented as the mean of five independent experiments.

### 4.6. Transwell Migration Assays

Serum-starved cells were recovered as single-cell suspensions, and 5 × 10^4^ cells were seeded in 0.5% serum growth media, with and without 100 ng/mL recombinant Noggin (PeproTech, London, UK), into the upper well of a transwell migration chamber (8 μm pore size; Corning, New York, NY, USA), pre-coated with fibronectin (10 μg/mL in PBS overnight at 4 °C). Migration was measured over 16 h by contacting the chambers with medium containing 0.5% serum at 37 °C. Following incubation, transwells were fixed in 30% methanol and stained with 1% crystal violet. Representative fields were photographed using an Axiovert 40CFL inverted microscope (Zeiss, Oberkochen, Germany), and relative rates of cell migration were determined by counting the number of stained cells.

### 4.7. Immunohistochemistry (IHC) and IHC Scoring

The expression of proteins of interest was assessed using standard immunohistochemical staining protocols and scored using a semi-quantitative system [50]. For each antibody examined, 10 NPC biopsy specimens containing normal adjacent epithelium (NPE) were scored for expression of BMP2 and phospho-SMAD1. Antibodies specific for BMP2 (ab6285; Abcam, Cambridge, UK) and phospho-SMAD1 (ab73211; Abcam, Cambridge, UK) were used at assay-dependent concentrations and used in a standard IHC protocol as previously described [50]. A semi-quantitative scoring system was used to evaluate IHC staining. Scores (values 0–9) were obtained by multiplying the staining intensity (negative = 0, weak = 1, moderate = 2, strong = 3) by the proportion of positive cells (<30% = 1, 30–70% = 2, >70% = 3).

### 4.8. Statistics

Where appropriate, statistical significance was calculated by performing a Student’s *t*-test having first determined equal or unequal variance by using an F-test.

## 5. Conclusions

Our study identified the presence of a prominent BMP “signature” in EBV-positive NPC, suggesting that aberrant BMP activation may contribute to the aetiology of this virus-associated cancer. Importantly, we showed that the genome maintenance protein, EBNA1, is the major viral-encoded protein responsible for activating the BMP pathway, through a mechanism involving autocrine induction of a BMP ligand. Collectively, this study supports a role for the BMP pathway in promoting cell migration and possibly, metastatic spread of this cancer.

## Figures and Tables

**Table 1:** RT-PCR primer sequences.

Gene	Sequence
ACVR1	Forward: GCTGCCCACTAAAGGAAAAT Reverse: GCGAGCCACTGTTCTTTGTA
ACVR2B	Forward: TCCCTCACGGATTACCTCA Reverse: CCTCCTCAAAAGGCAGCA
BMP2	Forward: CCTGAAACAGAGACCCACC Reverse: GCATTCTGATTCACCAACCT
BMPR1A	Forward: GTGGGTCTGGACTACCTT Reverse: GGGCACATCAACTTCATT
BMPR1B	Forward: CCACCCTAGACGCTAAAT Reverse: GCTCTCGTCCAACACTTCT
BMPR2	Forward: CCTGATGTTCTGCCTACT Reverse: GCTCTTCTGGGCTTTGAT
EBNA1	Forward: GGGTGGTTTGGAAAGCAT Reverse: TGGAAACCAGGGAGGCAAAT
GAPDH	Forward: GCCTCCTGCACCACCAACTG Reverse: CGACGCCTGCTTCACCACCTTCT
Smad1	Forward: CTCTCCCACCAGCTCAGA Reverse: CACTAAGGCATTCGGCAT
Smad5	Forward: CGCCTCCTCCTGCCTATA Reverse: GCTGCTGGGAATCTTACA
Smad9	Forward: CGCCTACTATGAACTGAA Reverse: GGAAGCCGTGTTGATAGT

## Supplementary Materials

The following are available online at https://www.mdpi.com/2076-0817/9/7/594/s1, Figure S1: Gene expression profiling of BMP pathway-associated genes in NPC tumours. Figure S2: Expression of EBNA1 at the RNA and protein levels in EBNA1-transfected and EBV-infected Ad/AH, HONE-1 and AGS cell lines. Figure S3: Expression of BMP pathway components in the Ad/AH, HONE-1 and AGS cell panels. Figure S4: The effect of inhibition of BMP signalling on the migration of Ad/AH, HONE-1 and AGS carcinoma cell lines. Figure S5: Potential crosstalk between TGFβ and BMP signalling pathways in Ad/AH, HONE-1 and AGS cells. Table S1: Fold change and *p*-values for BMP-associated genes differentially regulated between normal nasopharyngeal epithelium (NPE) and NPC tumours.
