# Supplementary material for: The Epstein-Barr Virus-Encoded EBNA1 Protein Activates the Bone Morphogenic Protein (BMP) Signalling Pathway to Promote Carcinoma Cell Migration
Source: Pathogens. 2020 Jul 21;9(7):594. doi: 10.3390/pathogens9070594 (PMC7400503; doi:10.3390/pathogens9070594)
Supplement: Supplementary file 1 [file pathogens-09-00594-s001.pdf]

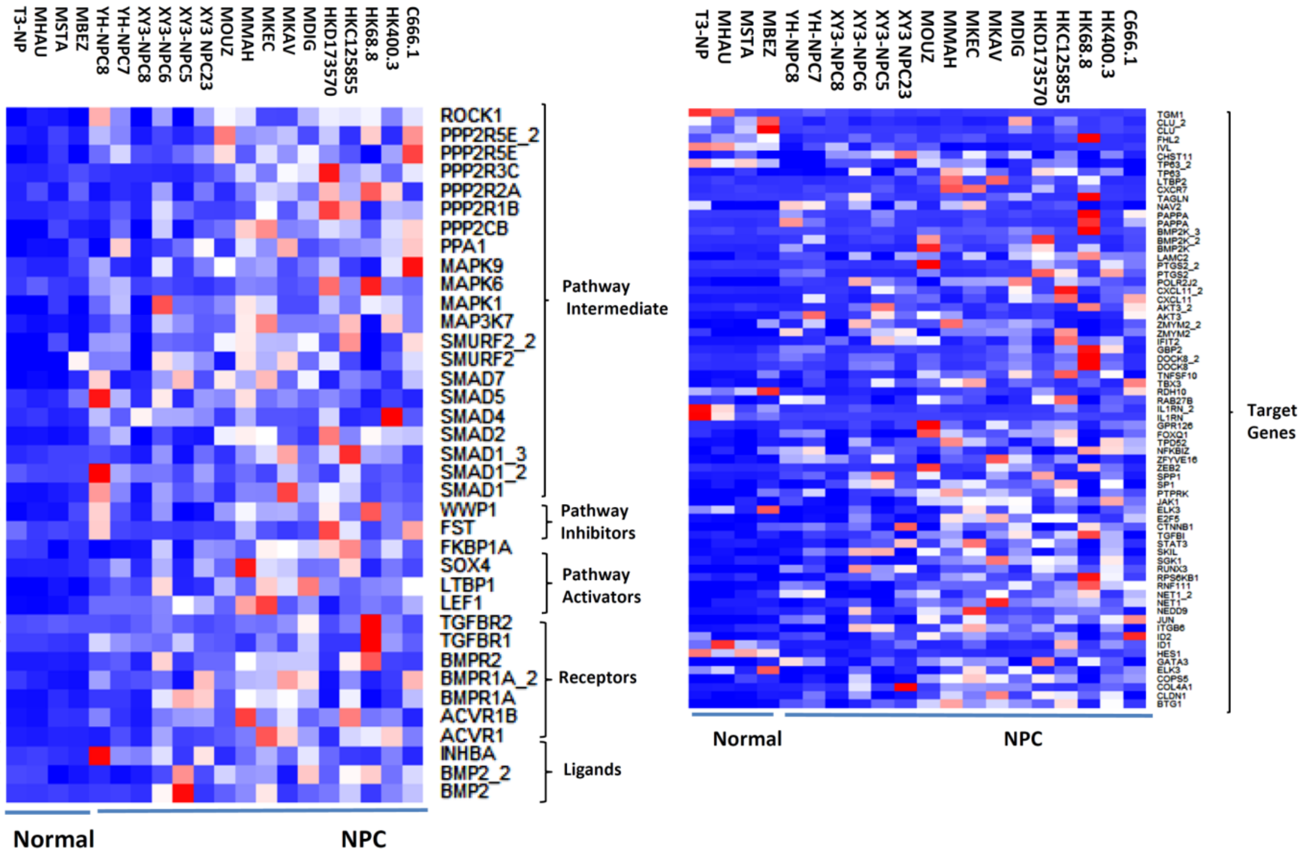

Figure S1: Gene expression profiling of BMP pathway-associated genes in NPC tumours. Extensive literature searches revealed a set of genes associated with the BMP signalling pathway. The resulting gene list was utilised to identify significantly differentially regulated genes, using expression array data generated in a separate study from 16 tumours and 4 normal controls, which were processed using dChip software and visualised using the heatmap package in R studio. The expression level of each gene in an individual sample is colour coded: blue for downregulation, red for upregulation and white for unchanged.

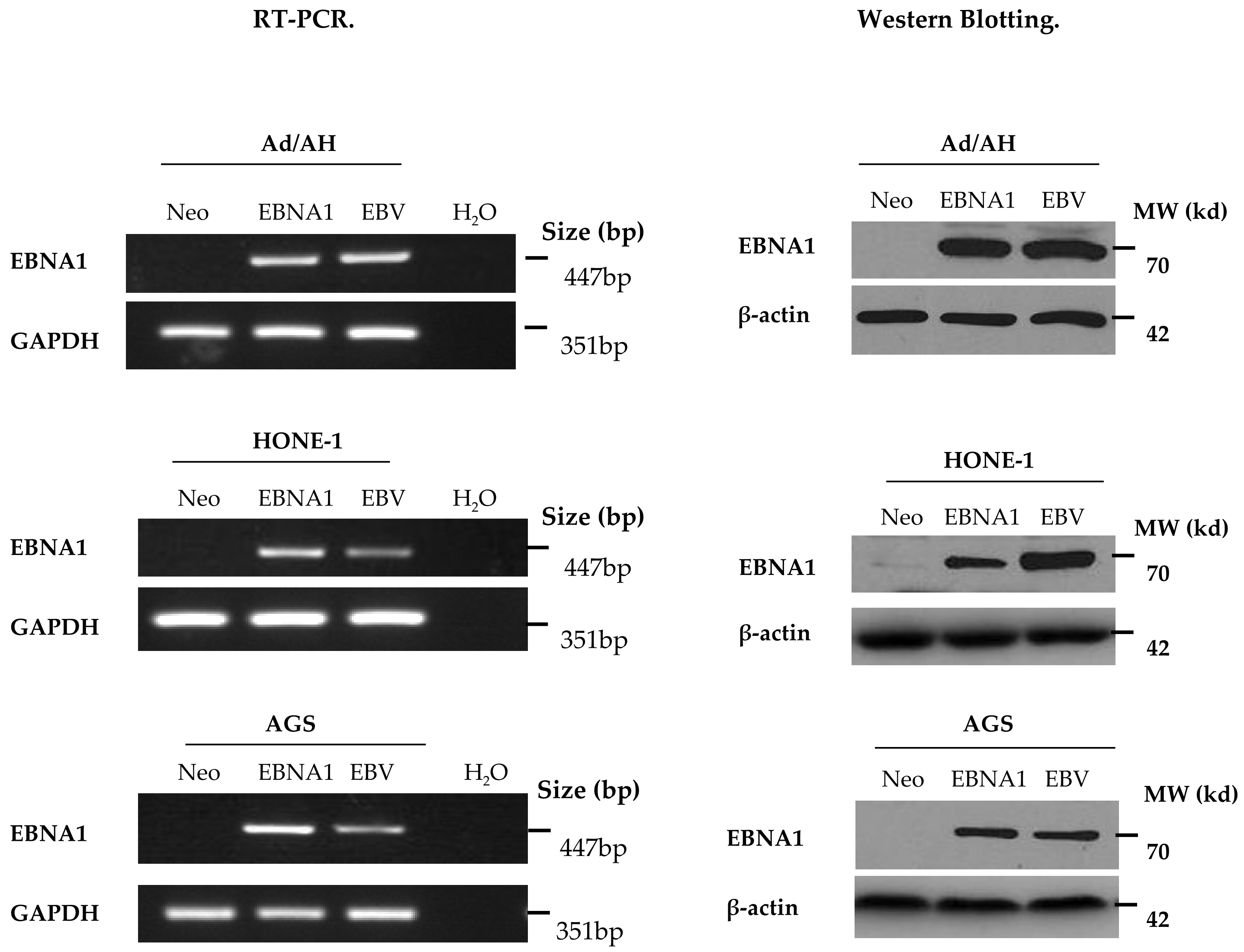

(b)

Figure S2: Validation of EBNA1 expression in the EBNA1 transfected and EBV infected carcinoma cell lines. RT-PCR (left panels) and western blotting (right panels) confirming EBNA1 expression in Ad/AH, HONE-1 and AGS cell lines stably expressing EBNA1 or latently infected with EBV. RT-PCR analysis was performed on mRNA extracted from neomycin control, EBNA1 transfected or EBV infected cell lines from each cell panel. H_20 was included as a negative control. Western blotting of total cell lysates was performed using K67, 1:1000, a rabbit anti-serum, which detects EBNA1. β-actin was included to confirm equal protein loading.

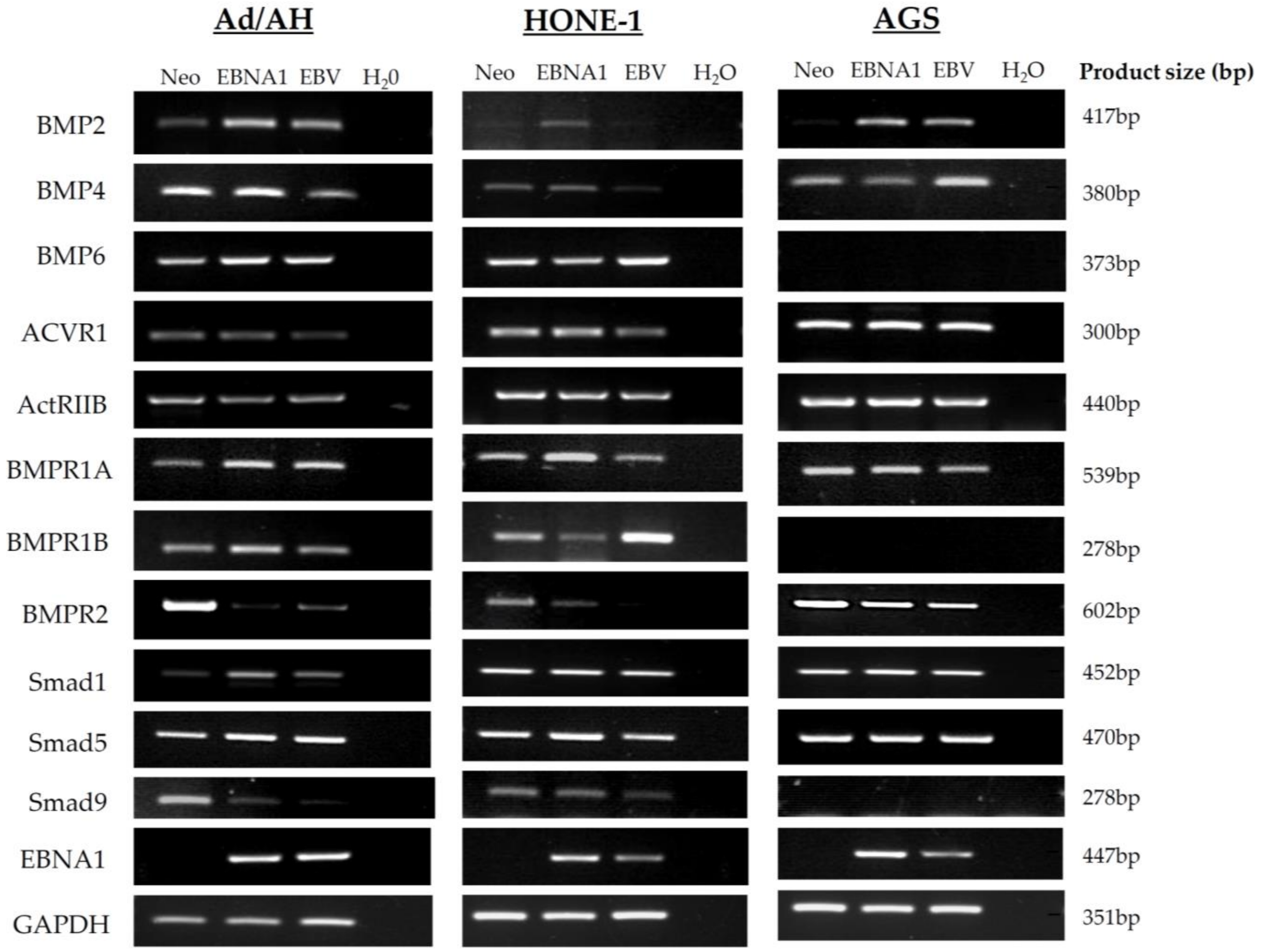

Figure S3: Expression of BMP pathway components in the Ad/AH, HONE-1 and AGS cell panels. RT-PCR analysis for mRNA expression levels of BMP ligands (BMP2, 4, 6 and 7), BMP receptors (ActRI, ActRIIB, BMPRIA, BMPRIB and BMPRII), BMP-specific Smads (Smad1, Smad5 and Smad8) and classical BMP target genes (Id1, Id2 and Id3) in the Ad/AH, HONE-1 and AGS cell panels. EBNA1 was included as a control for EBV expression in the EBNA1 and EBV-infected cell lines and GAPDH was included as a positive control to confirm equal RNA input into the PCR reactions, while negative water controls confirmed the absence of contamination.

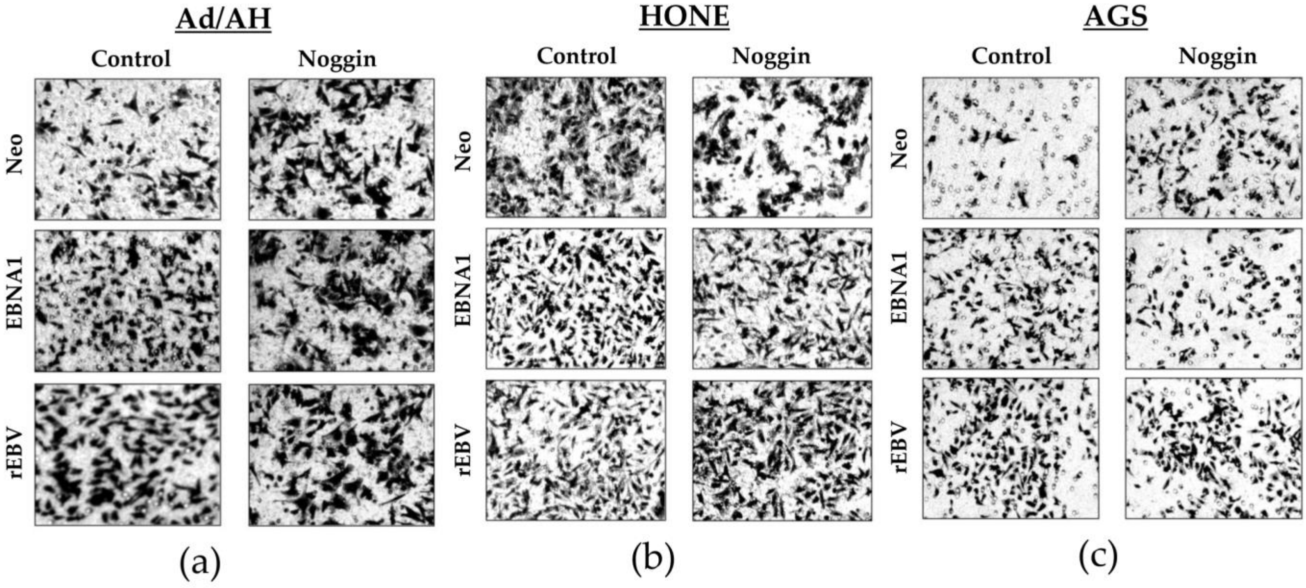

Figure S4. The effect of inhibition of BMP signaling on the migration of Ad/AH, HONE-1 and AGS carcinoma cell lines. Serum-starved Ad/AH (A), HONE-1 (B) and AGS (C) cells expressing either a control neomycin resistance cassette or EBNA1, or stably infected with rEBV were seeded into the upper wells of transwell migration chambers in serum-free medium with and without 100 ng/mL Noggin, and allowed to migrate for 24 hours. The number of migrated cells in five representative fields were counted and a representative image is shown.

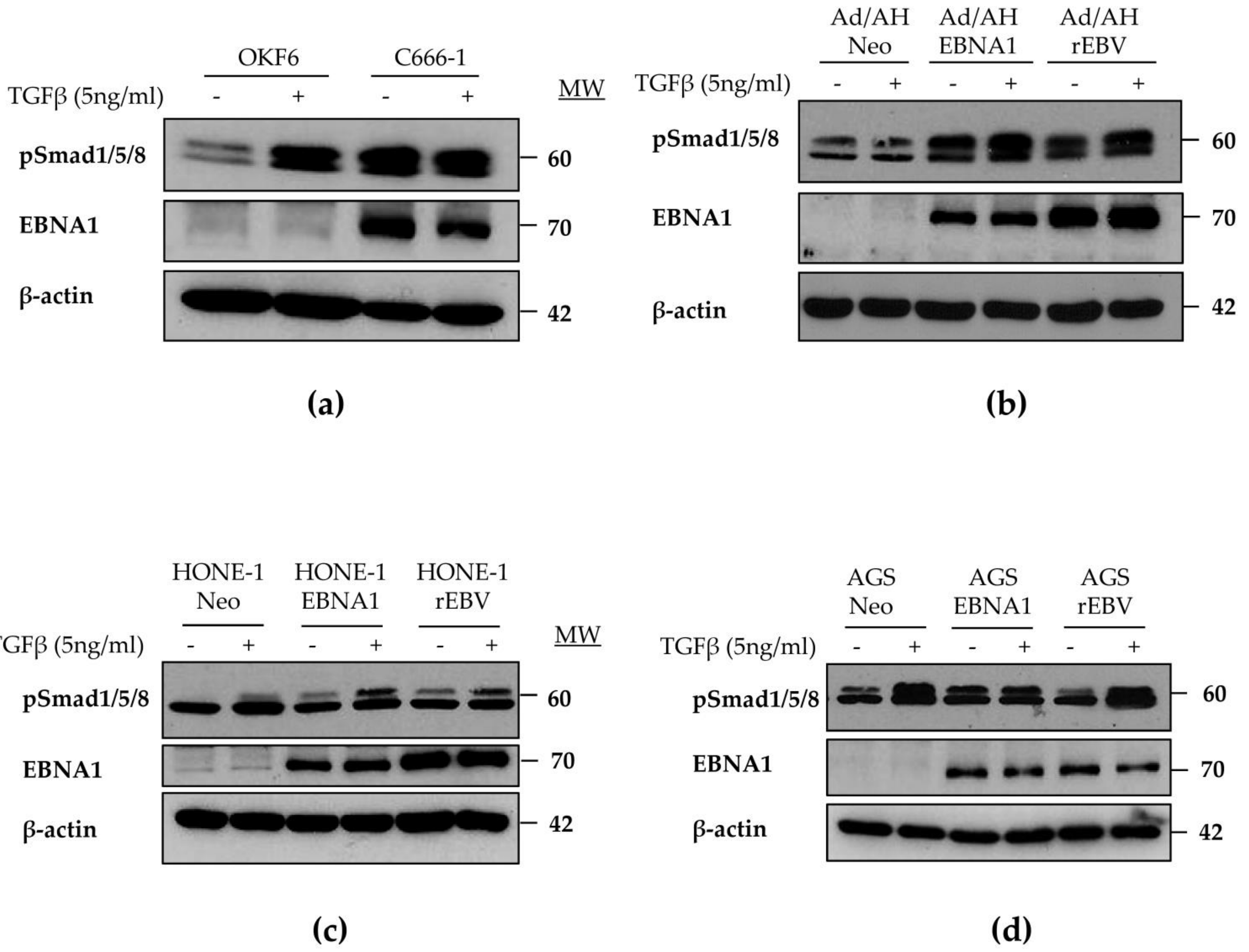

Figure S5. Potential crosstalk between TGFβ and BMP signalling pathways in Ad/AH, HONE-1 and AGS cells. Immunoblotting for protein levels of the phosphorylated form of Smad1/5/8 in serum-starved (a) OKF6 and C666-1 cells, (b) Ad/AH (c), HONE-1 (d) and AGS cells expressing either a control neomycin resistance cassette or EBNA1, or stably infected with rEBV, stimulated with 5ng/ml recombinant TGFβ1 for 24 hours. Immunblotting for EBNA1 was included for confirmation of cell line status and blots were re-probed for β-actin to confirm equal protein loading.

Supplementary Table 1. Fold change and p-values for BMP-associated genes differentially regulated between four normal nasopharyngeal epithelium (NPE) and sixteen NPC tumours. Compiled from Hu *et al.*, 2012 [50].

	Probe Set	Description	Fold change FC:(class1/class2)	p-value. (t)
**Ligands**	205290_s_at	bone morphogenetic protein 2	2.4	0.00135796
	210511_s_at	inhibin, beta A	2.8	0.00277305
**Receptors**	203935_at	activin A receptor, type I	5.2	8.1589E-06
	213198_at	activin A receptor, type IB	2.9	0.00229758
	204832_s_at	bone morphogenetic protein receptor, type IA	4.0	0.00276395
	225144_at	bone morphogenetic protein receptor, type II (serine/threonine kinase)	5.8	3.4503E-07
	224793_s_at	transforming growth factor, beta receptor 1	3.1	0.00010517
	208944_at	transforming growth factor, beta receptor II (70/80kDa)	2.1	0.05681665
**Pathway Activators**	226853_at	BMP2 inducible kinase	2.78	0.00177154
	221558_s_at	lymphoid enhancer-binding factor 1	4.8	0.00028056
	202729_s_at	latent transforming growth factor beta binding protein 1	4.6616	6.4648E-05
	201417_at	SRY (sex determining region Y)-box 4	14.2947	0.00136784
**Pathway Inhibitors**	200709_at	FK506 binding protein 1A, 12kDa	1.91	0.04253026
	226847_at	follistatin	2.0229	0.21408886
	212638_s_at	WW domain containing E3 ubiquitin protein ligase 1	3.7301	0.02392665
**Pathway Intermediates**	227798_at	SMAD family member 1	5.1	0.00072617
	203077_s_at	SMAD family member 2	3.5	5.621E-05
	1565703_at	SMAD family member 4	3.2	0.00016441
	225219_at	SMAD family member 5	2.4	0.13049429
	204790_at	SMAD family member 7	4.4	0.00295922
	205596_s_at	SMAD specific E3 ubiquitin protein ligase 2	3.0629	0.1687603
	206854_s_at	mitogen-activated protein kinase kinase kinase 7	4.3653	8.6932E-05
	224621_at	mitogen-activated protein kinase 1	2.3902	0.00454922
	207121_s_at	mitogen-activated protein kinase 6	2.1275	0.02254171

	203218_at	mitogen-activated protein kinase 9	2.28	0.00650559
	217848_s_at	pyrophosphatase (inorganic) 1	1.9637	0.00982212
	201375_s_at	protein phosphatase 2, catalytic subunit, beta isozyme	2.8445	0.02048075
	202883_s_at	protein phosphatase 2, regulatory subunit A, beta	1.9489	0.00184979
	202313_at	protein phosphatase 2, regulatory subunit B, alpha	2.0281	0.14629313
	218852_at	protein phosphatase 2, regulatory subunit B'', gamma	3.4824	0.02503014
	203338_at	protein phosphatase 2, regulatory subunit B', epsilon isoform	3.4508	0.00864222
	213044_at	Rho-associated, coiled-coil containing protein kinase 1	2.2446	0.00130645
**Pathway Targets**	200921_s_at	B-cell translocation gene 1, anti-proliferative	14.1825	0.02812073
	222549_at	claudin 1	2.6721	0.01066269
	211980_at	collagen, type IV, alpha 1	10.4815	0.00134049
	201652_at	COP9 constitutive photomorphogenic homolog subunit 5 (Arabidopsis)	1.8122	0.13774332
	209604_s_at	GATA binding protein 3	2.7102	0.00200907
	218469_at	GREM1, cysteine knot superfamily, homolog (Xenopus laevis)	2.42	0.00303
	203394_s_at	hairy and enhancer of split 1, (Drosophila)	-5.720823799	2.8209E-05
	208937_s_at	inhibitor of DNA binding 1, dominant negative helix-loop-helix protein	-3.680529996	0.01954223
	201565_s_at	inhibitor of DNA binding 2, dominant negative helix-loop-helix protein	1.8458	0.21410348
	226535_at	integrin, beta 6	3.6031	0.10655453
	201466_s_at	jun proto-oncogene	4.9458	0.00663596
	202149_at	neural precursor cell expressed, developmentally down-regulated 9	4.6487	0.00763948
	201830_s_at	neuroepithelial cell transforming 1	2.3812	0.05250833
	218761_at	ring finger protein 111	4.2177	0.00265316
	204171_at	ribosomal protein S6 kinase, 70kDa, polypeptide 1	1.9665	0.003246883
	213044_at	Rho-associated, coiled-coil containing protein kinase 1	2.24	0.00130645

	204197_s_at	runt-related transcription factor 3	2.77	0.000823894
	201739_at	serum/glucocorticoid regulated kinase 1	2.0286	0.04658807
	215889_at	SKI-like oncogene	2.65	0.00015704
	201417_at	SRY (sex determining region Y)-box 4	14.2947	0.00136784
	208991_at	signal transducer and activator of transcription 3 (acute-phase response factor)	2.51	0.005005632
	201506_at	transforming growth factor, beta-induced, 68kDa	2.03	0.02285851
	223679_at	catenin (cadherin-associated protein), beta 1, 88kDa	2.48	0.05800336
	221586_s_at	E2F transcription factor 5, p130-binding	2.7893	0.00086285
	221773_at	ELK3, ETS-domain protein (SRF accessory protein 2)	-2.612330199	0.1160571
	201648_at	Janus kinase 1	3.18	0.02363063
	203038_at	protein tyrosine phosphatase, receptor type, K	4.337	0.10865259
	224754_at	Sp1 transcription factor	1.9111	0.01624151
	209875_s_at	secreted phosphoprotein 1	5.0	0.00030636
	203603_s_at	zinc finger E-box binding homeobox 2	2.00	0.00761306
	203651_at	zinc finger, FYVE domain containing 16	2.4803	0.00901215
	223218_s_at	nuclear factor of kappa light polypeptide gene enhancer in B-cells inhibitor, zeta	2.16	0.221119
	201690_s_at	tumor protein D52	2.61	0.00379873
	227475_at	forkhead box Q1	2.09	0.03673839
	213094_at	G protein-coupled receptor 126	2.18	0.0033964
	212657_s_at	interleukin 1 receptor antagonist	-10.11	0.02617288
	228708_at	RAB27B, member RAS oncogene family	1.9	0.10634818
	1552378_s_at	retinol dehydrogenase 10 (all-trans)	-4.73	0.0039796
	219682_s_at	T-box 3	2.8	0.00047188
	202688_at	tumor necrosis factor (ligand) superfamily, member 10	2.59	0.17155074
	225502_at	dedicator of cytokinesis 8	5.39	0.01351608
	202748_at	guanylate binding protein 2, interferon-inducible	2.28	0.00632079

	226757_at	interferon-induced protein with tetratricopeptide repeats 2	8.56	2.51E-05
	210282_at	zinc finger, MYM-type 2	6.14	0.03015924
	224229_s_at	v-akt murine thymoma viral oncogene homolog 3 (protein kinase B, gamma)	5.51	0.00036601
	210163_at	chemokine (C-X-C motif) ligand 11	4.97	6.07E-05
	1552621_at	polymerase (RNA) II (DNA directed) polypeptide J2	4.29	0.00257493
	204748_at	prostaglandin-endoperoxide synthase 2 (prostaglandin G/H synthase and cyclooxygenase)	2.99	0.00189618
	202267_at	laminin, gamma 2	2.95	0.05186757
	212764_at	zinc finger E-box binding homeobox 1	2.82	0.000844
	226853_at	BMP2 inducible kinase	2.78	0.00177154
	228128_x_at	pregnancy-associated plasma protein A, pappalysin 1	2.70	0.01173797
	218330_s_at	neuron navigator 2	2.66	0.2213328
	205547_s_at	transgelin	2.04	0.06872712
	212977_at	chemokine (C-X-C motif) receptor 7	2.00	0.12313027
	223690_at	latent transforming growth factor beta binding protein 2	1.90	0.0556189
	211834_s_at	tumor protein p63	-3.40	3.94E-05
	226368_at	carbohydrate (chondroitin 4) sulfotransferase 11	1.71	0.17721501
	214599_at	involucrin	-2.53	0.00051036
	202949_s_at	four and a half LIM domains 2	-2.53	0.02562237
	208792_s_at	clusterin	-5.31	0.0424232
	206008_at	transglutaminase 1 (K polypeptide epidermal type I, protein-glutamine-gamma-glutamyltransferase)	-3.76	0.10694314
